# Isolation, Characterization, and Depolymerization of l‐Cysteine Substituted *Eucalyptus* Lignin

**DOI:** 10.1002/gch2.202100130

**Published:** 2022-03-03

**Authors:** Lanlan Shi, Tanhao Zhang, Xin Zhou, Lu Yao, Linjie Yang, Fengxia Yue, Wu Lan, Fachuang Lu

**Affiliations:** ^1^ State Key Laboratory of Pulp and Paper Engineering School of Light Industry and Engineering South China University of Technology Guangzhou 510640 China; ^2^ Department of Biochemistry and Great Lakes Bioenergy Research Center The Wisconsin Energy Institute University of Wisconsin Madison WI 53726 USA

**Keywords:** 2D NMR, condensation, l‐cysteine lignin, model compounds

## Abstract

Lignin condensation reactions are hard to avoid or control during separation, which is a deterrent to lignin isolation and post‐conversation, especially for the full utilization of lignocelluloses. Selective protection of β‐aryl ether linkages in the isolation process is crucial to lignin valorization. Herein, a two‐step acid/alkali separation method assisted with l‐cysteine for *eucalyptus* lignin separation is developed, and the isolated l‐cysteine lignins (LCLs) are comprehensively characterized by 2D NMR, ^31^P NMR, thioacidolysis, etc. Compared to the two‐step control treatment, a much higher β‐O‐4 content is preserved without reducing the separation efficiency assisted by l‐cysteine, which is also significantly higher than alkali lignin and kraft lignin. The results of hydrogenolysis show that LCLs generate a much higher monomer yield than that of control sample. Structural analysis of LCLs suggests that lignin condensation reaction, to some extent, is suppressed by adding l‐cysteine during the two‐step acid/alkali separation. Further, mechanistic studies using dimeric model compound reveals that l‐cysteine may be the α‐carbon protective agent in the two‐step separation. The role of l‐cysteine in the two‐step lignin isolation method provides novel insights to the selective fractionation of lignin from biomass, especially for the full valorization of lignocellulosic biomass.

## Graphical abstract

An organic solvent‐free and toxic chemical‐free separation method assisted by l‐cysteine is developed for lignin isolation, and the obtained lignins are comprehensively evaluated by structural characterization and depolymerization, revealing the role of l‐cysteine as an α‐C protective agent to suppress the cleavage of β–O–4 in lignin fragments in the two‐step separation.

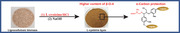

## 1 Introduction

With the growing of global energy and climate issues, conversion of lignocellulosic biomass into bioethanol, biodiesel, renewable jet fuel, and platform chemicals has drawn more and more attentions.^[^ ^1^, ^2^, ^3^, ^4^, ^5^ ^]^ Efficient conversion of lignocellulosic biomass, to a certain extent, can not only alleviate the dependence on fossil energy, but also benefit the environmental protection and greenhouse gas emission reduction, which is of great importance to green and sustainable economic development.^[^ ^6^, ^7^, ^8^, ^9^ ^]^ Lignin, a major component of lignocellulosic biomass accounted for 15–35 wt% (varies from plant species and tissues), is the most abundant aromatic resource on the earth yet highly underutilized.^[^ ^10^, ^11^ ^]^ In general, the primary goal of traditional lignocellulose biorefinery is to get the value products from the carbohydrate fractions, especially cellulose. Therefore, lignocellulosic biomass fractionation or pretreatment is always under solvolysis, acidic and basic conditions with high temperatures and/or inexpensive mineral acids (such as HCl and H_2_SO_4_) to remove lignin and hemicellulose, especially for the pulping and paper industry.^[^ ^12^, ^13^, ^14^, ^15^ ^]^ However, it can not only cleave the lignin ether bonds but also lead to the new carbon—carbon (C—C) bonds formation, which is an irreversible condensation reaction. This leads to the difficulty of lignin valorization, particularly for the depolymerization to aromatic chemicals.^[^ ^11^, ^16^, ^17^ ^]^

In recent years, many efforts have been donated to lignin stabilization during the lignin fractionation or degradation, and remarkable progresses were made.^[^ ^9^, ^11^, ^18^, ^19^, ^20^ ^]^ An important strategy is trapping reactive intermediates to stabilize lignin, which has attracted enormous attention ever since it was proposed. For example, several kinds of aldehydes including formaldehyde and acetaldehyde, were applied to react with the α, γ‐diol on the side chain of lignin by forming a cyclic acetal structure to prevent undesirable condensation or repolymerization, which have greatly promoted the lignin‐derived monomer yields during biomass depolymerization.^[^ ^21^, ^22^ ^]^ Besides, another class of methods was developed to suppress lignin condensation , including using deep eutectic solvent, ionic liquids, organic acid catalyst, *p*‐toluene sulfonic acid, acidic lithium bromide trihydrate, etc., as the solvents or catalysts to extract lignin.^[^ ^23^, ^24^, ^25^, ^26^, ^27^ ^]^ Among these methods, alcohols (methanol, ethanol, and butanol) extraction was demonstrated to be a feasible method to isolate lignin and meanwhile protect the integrity of the β‐O‐4 linked structure through incorporating at the benzylic α‐position.^[^ ^28^, ^29^ ^]^ Compared to kraft lignin and alkali lignin, those lignins isolated under mild conditions or using protection reagents result in a higher reactivity toward subsequent valorization process.^[^ ^30^, ^31^, ^32^ ^]^

In the early report of lignin quantification, thioglycolic acid was applied to swell and dissolve lignin. Lignin derivatization was happened by the reaction of thioglycolic acid with the α carbocation on the side chain of β‐O‐4 linkage, and the dissolved lignin was then quantified using ultraviolet spectrophotometry.^[^ ^33^ ^]^ But the unbearable odor of thioglycolic acid limits its wide application. l‐cysteine is an amino acid bearing a thiol group. Considering the similar functional groups between thioglycolic acid and l‐cysteine, as well as the high safety to environment and human being, l‐cysteine shows potential in lignin derivation and isolation. Recently, our group developed a facile spectroscopic method for quantitation of the lignin in lignocellulosic material using l‐cysteine to promote the complete dissolution of lignin.^[^ ^34^ ^]^

Based on the previous study, we attempted to apply l‐cysteine in lignin isolation. We designed a two‐step acid/alkali separation method assisted with l‐cysteine for *eucalyptus* lignin separation, in which the first step is aiming to modify lignin with l‐cysteine and the second step is aiming to dissolve the derivative lignin. The isolated lignin preparations were characterized by elemental analysis, thioacidolysis, ^31^P NMR, and 2D NMR spectroscopy. The possible reaction mechanism or the role of l‐cysteine was revealed by synthetic model compound study. Additionally, the ability of l‐cysteine lignin (LCL) hydrogenolysis to generated phenolic monomer compounds was also explored.

## 2 Results and Discussion

### 2.1 Chemical Composition Analysis

**Figure 1:**
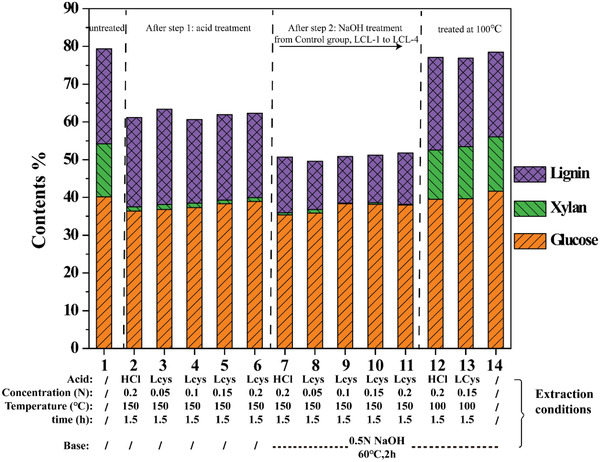
Composition analysis of *eucalyptus* residue under different conditions. Note: Lcys means l‐cysteine; /: untreated.

The changes of lignocellulosic biomass composition during the separation process can be an important evaluation index for the effect of the treatment method.^[^ ^35^, ^36^ ^]^ Chemical components of eucalyptus treated under different conditions are shown in **Figure** **1**. The contents of glucose, xylose, and lignin of untreated eucalyptus powder are 40.1%, 14.1%, and 25.1%, respectively (entry 1, Figure 1). By comparison, significant changes occurred after the sequential two‐step treatment of eucalyptus powder. The yields of the solid residue were about 50% via the sequential acid/alkali two‐step treatment (entry 7–11) based on raw material. It can be seen that the three main compositions, including glucan, xylan, and lignin, were all reduced either in the stage of acid pretreatment or the alkali extraction. However, there is a big difference between the two stages, for instance, 88.9–93.0% hemicellulose was removed in the dilute acid treatment stage (entry 2–6), whereas delignification was mainly occurred in the alkali extraction stage (entry 7–11). Meanwhile, the treatment at low temperature (100 °C; entry 12 and 13) or only under the base condition (0.5 N NaOH; entry 14) only led to a slight decrease of lignin and hemicellulose.

The different contents of the solid residues under different conditions were mainly caused by the different roles between the dilute acid and alkali on lignocellulosic biomass degradation. As shown in Figure 1, hemicellulose was easy to be removed under the acid treatment stage, while lignin showed a certain resistance to acid and alkali treatment. Moreover, the removal of lignin is 41.4–49.2%,^[^ ^37^ ^]^ which could be owing to the low temperature (60 °C) and short incubation time (2 h) during the alkali process. In addition, most of the cellulose was retained after the two‐step treatment, which indicated a possible way for lignocellulose component fractionation.

### 2.2 Elemental Analysis

The results of elemental analysis are shown in **Table** **1**. As shown in the control sample, trace amount of nitrogen and sulfur could be observed in lignin preparation, which could be derived from the residual protein in the lignin.^[^ ^38^ ^]^ By comparison, the contents of nitrogen and sulfur, especially the sulfur content, in the lignin isolated with l‐cysteine were increased evidently, suggesting that lignin could be modified directly by reaction with l‐cysteine during the sequential two‐step fractionation process. The content of N plus O was slightly lower than the theoretical value, which may be ascribed to the deamination and decarboxylation.

**Table 1:** The contents of C, H, N, and S elements in l‐cysteine lignins

Sample	Element contents [%]			
	C	H	N	S
Control	58.86	5.34	0.09	0.01
LCL‐1	52.24	4.99	0.56	2.48
LCL‐2	56.02	5.56	0.93	4.22
LCL‐3	54.46	5.62	1.02	5.37
LCL‐4	53.91	5.59	1.05	6.69

### 2.3 Molecular Weight

**Figure 2:**
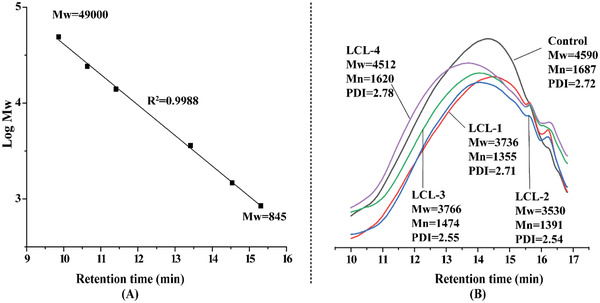
The calibration curve for A) lignin molecular weight calculation and B) the chromatogram of the LCL in gel‐permeation chromatography.

The molecular weight analysis of different lignin preparations was measured by the gel‐permeation chromatography (GPC) and is shown in **Figure** **2**. Nine reference polystyrene samples with a weight‐average molecular weight in the range of 208–49 000 g mol^−1^ were used for the molecular weight calibration of fractional lignins (Figure 2A). The weight‐average molecular weight (*M*_w_) and number‐average molecular weight (*M*_n_) of the lignin preparations are shown in Figure 2B. Without using l‐cysteine, the *M*_w_ of isolated lignin was 4590. However, the molecular weights of LCL samples were 3736 (LCL‐1), 3530 (LCL‐2), 3766 (LCL‐3), and 4512 (LCL‐4), respectively. It can be seen that the *M*_w_ of LCLs, especially the LCL‐1, LCL‐2, and LCL‐3, were lower than the control sample. It is speculated that the addition of l‐cysteine could prevent the condensation of lignin during isolation process, which could be further proved by the NMR characterization. Additionally, the increased *M*_w_ of LCL‐4 could attribute to the grafting of l‐cysteine onto the lignin, leading to increased amount of carboxylate groups, thereby increasing the solubility of lignin fragments with higher *M*_W_ compared to the other conditions. This was similar to the result of alcohol extraction of lignin.^[^ ^28^ ^]^

### 2.4 ^31^P NMR Analysis

**Figure 3:**
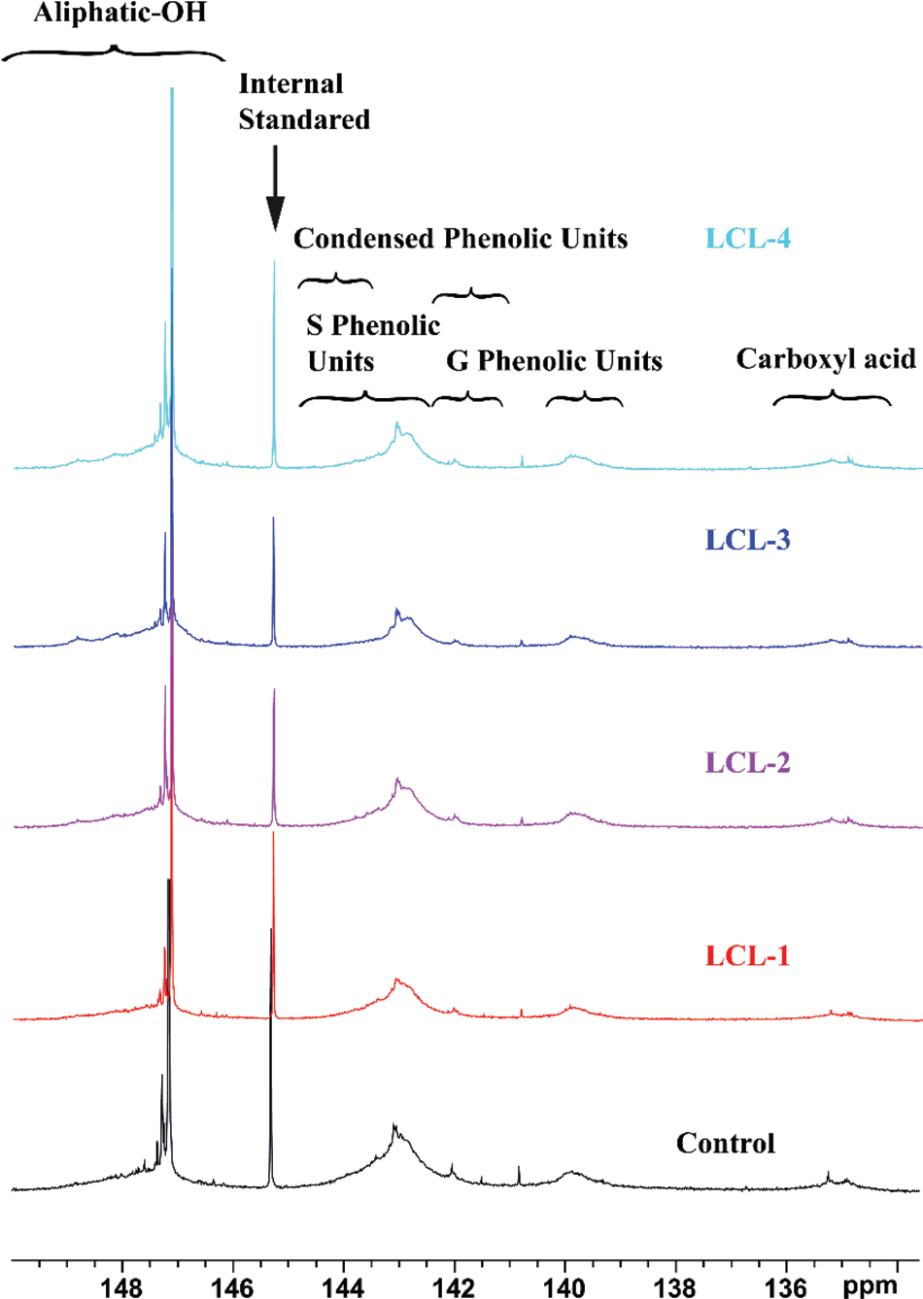
Quantitative ^31^P NMR spectra of LCLs.

^31^P NMR method is widely applied to quantify the hydroxyl group in lignin and lignin‐derived products.^[^ ^39^ ^]^ The ^31^P NMR spectra of isolated lignin samples are displayed in **Figure** **3** and the calculated hydroxyl contents are shown in **Table** **2**. According to the results shown in Table 2, the contents of aliphatic hydroxyl group and carboxyl acid of LCL‐4 lignin were 2.70 and 0.37 mmol g^−1^, respectively, which was increased by 59% and 61% based on the control sample. Besides, the content of condensed hydroxyl group in LCL‐4 lignin, including S‐OH and G‐OH, was reduced by 33.3%. The increment of hydroxyl groups, especially the phenolic groups indicated less condensation of LCL lignins than the control sample.

**Table 2:** The content and distribution of hydroxyl groups in LCL samples quantified by ^31^P NMR

Samples	Control	LCL‐1	LCL‐2	LCL‐3	LCL‐4
Aliphatic OH	1.70	1.79	2.05	2.69	2.70
Condensed S phenolic OH	0.61	0.64	0.52	0.36	0.43
Noncondensed S‐OH	0.85	0.94	0.97	0.90	0.84
Condensed G‐OH	0.29	0.25	0.23	0.17	0.17
Noncondensed G‐OH	0.35	0.37	0.40	0.36	0.37
Total phenol OH	2.10	2.20	2.12	1.80	1.80
Total OH	3.80	3.99	4.17	4.49	4.50
Carboxyl acid	0.23	0.26	0.29	0.34	0.37

### 2.5 Thioacidolysis Analysis

Being the most abundant interunit linkage in native lignin, β‐O‐4‐ether content is very essential index for lignin's properties. As shown in **Table** **3** (entry 1), the total lignin‐derived monomer yield of eucalyptus was 2590.2 µmol g^−1^ based on lignin content, including 972.9 µmol g^−1^ of guaiacyl unit and 1617.3 µmol g^−1^ of syringyl unit, and the S/G ratio was 1.66. The monomer yields of lignin were sharply decreased after isolated from eucalyptus via two‐step fraction with/or without using l‐cysteine, and the S/G ratios were changed accordingly as shown in Table 3 (entry of 2–8). The results suggested that a significant amount of β‐O‐4 linkages was cleaved when isolating lignin from lignocellulosic biomass due to the serve conditions used. However, compared the entry 2 to entry 3–6, the total monomer yield of the control lignin was 137.6 µmol g^−1^, which was accounted to 5.3% based on raw materials. Whereas the total monomer yield increased 2.33–4.05 times when applying l‐cysteine to assist the separation. It suggests that although it is hard to avoid condensation of lignin, more β‐O‐4 ether structure could be retained by using the l‐cysteine‐assisted two‐step process.

**Table 3:** The yields (µmol g^−1^ of lignin) of lignin‐derived thioacidolysis monomer from different lignin samples

Entry	Lignin	Monomer yields^a)^			Molar ratios
		G	S	Total yield	S/G
1	*Eucalyptus*	972.9	1617.3	2590.2	1.66
2	Control	33.2	104.4	137.6	3.15
3	LCL‐1	92.5	228.6	321.1	2.47
4	LCL‐2	94.9	268.7	363.6	2.83
5	LCL‐3	138.5	402.9	541.5	2.91
6	LCL‐4	134.9	415.7	550.6	3.08
7	AL^b)^	152.7	247.8	400.5	1.62
8	KL^c)^	14.6	23.3	37.9	1.60

^a)^ Yield (µmol g^−1^ of lignin) of *eucalyptus* raw material was calculated based on Kalson lignin, while other lignins were based on the lignin

^b)^ Eucalyptus alkali lignin (AL) obtained with condition: NaOH dosage 22%, liquor ratio 4.5, 162 °C for 40 min and raise to 170 °C in 20 min and hold 50 min^[^ ^40^ ^]^

^c)^ Kraft lignin obtained from Yunnan Yunjing forestry and paper industry Limited by Share Ltd. (Jinggu, Yunnan).

### 2.6 2D Heteronuclear Singular Quantum Correlation (HSQC) NMR

**Figure 4:**
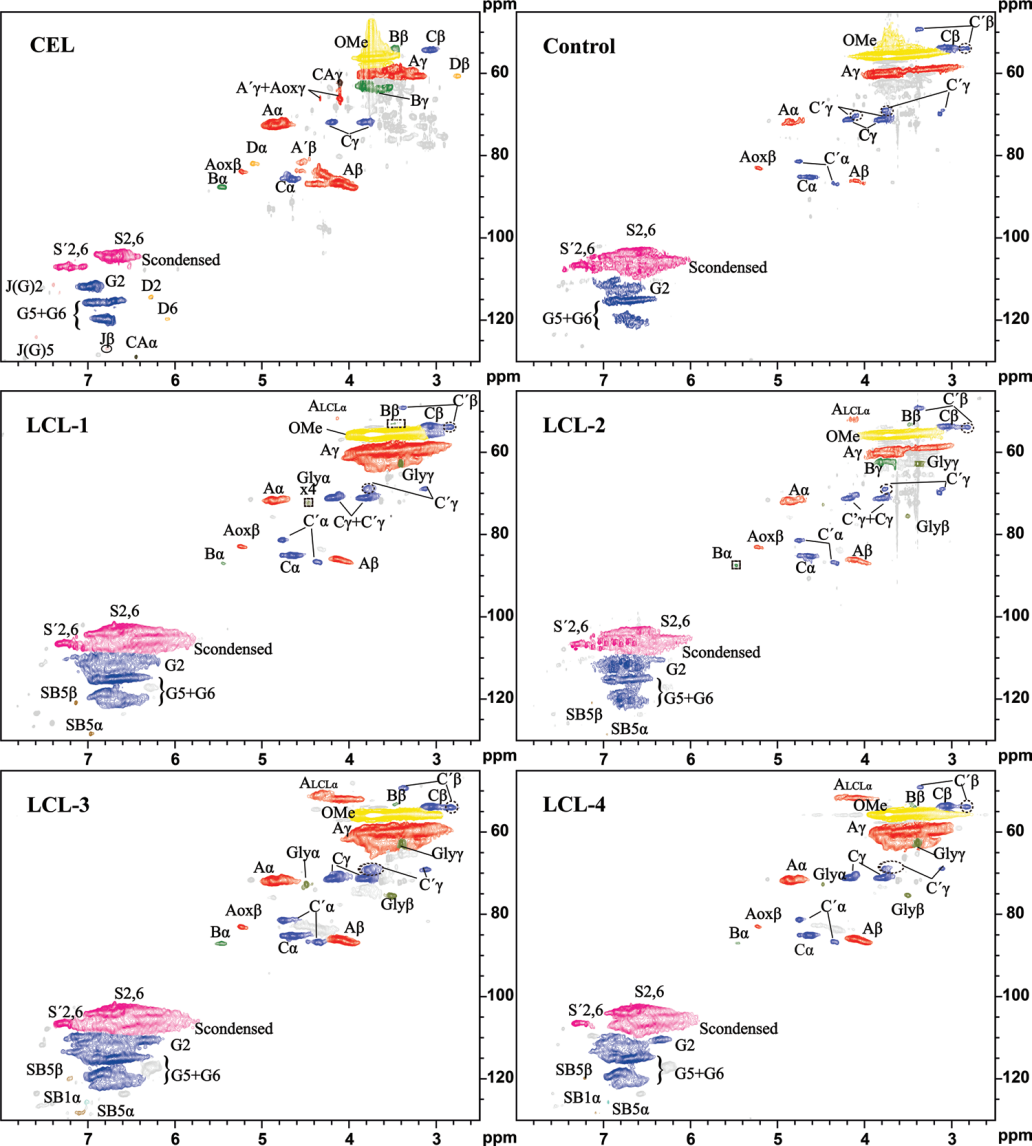
2D HSQC NMR spectra (DMSO‐*d*_6_) of different lignin samples (600 MHz).

**Figure 5:**
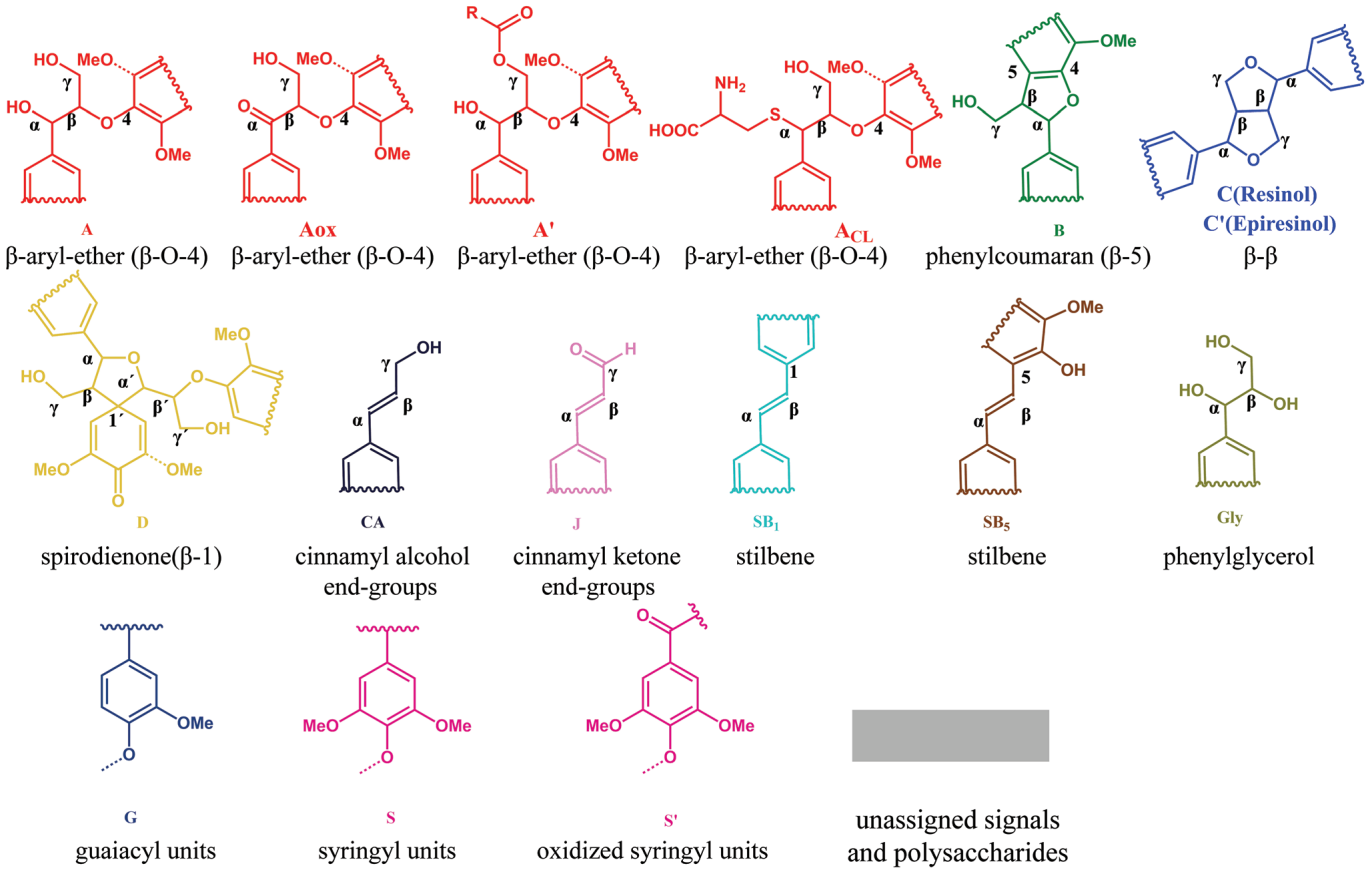
Substructures of isolated lignin corresponding to the HSQC spectra.

For better understanding the structural information, 2D HSQC NMR study was performed on the control and l‐cysteine‐substituted lignin, as well as the cellulolytic enzyme lignin (CEL), and the resulting and structure spectra are shown in **Figures** **4** and **5**. The main linkages were similar for LCLs and CEL, except the LCLs displayed the characteristic peaks of the β‐O‐4 linkage with l‐cysteine modified at Cα. The relative abundance of different interunit linkages or functional groups was calculated by integration of the corresponding contours (**Table** **4**).

**Table 4:** Percentages of the substructures in the isolated lignin samples determined by integration of the contours in the HSQC spectra

	Samples					
Structure^a)^	CEL	Control	LCL‐1	LCL‐2	LCL‐3	LCL‐4
Aromatic region						
SB1		<1%	<1%	<1%	<1%	<1%
SB5		<1%	<1%	<1%	<1%	<1%
S	62%	35%	36%	57%	64%	81%
S_condensed_	1%	56%	35%	30%	23%	10%
S'	9%	2%	2%	4%	2%	1%
G	28%	8%	14%	8%	11%	8%
Aliphatic side chain region						
Aox	3%	–	1%	1%	1%	1%
A	57%	3%	5%	12%	12%	13%
A'	4%					
A_LCL_			<1%	1%	7%	12%
B	3%		0%	1%	1%	1%
C	12%	5%	2%	9%	7%	9%
C'		3%	1%	5%	4%	5%
Gly	–	–	2%	2%	1%	2%
Total β‐O‐4	64%	3%	6%	14%	20%	25%

^a)^ The percentages of the substructures in the aromatic region and the interlinkages in the side chain region in the HSQC spectra were calculated according to pervious report.^[^ ^41^ ^]^ C2/6‐H2/6 correlations (divided by 2) from (S+S') units, C6‐H6 correlations from S_condensed_ units, and C2‐H2 correlation from G units were counted as 100%, and other substructures were calculated based on it. In addition, total β‐O‐4 (AT) ratios represent the percentages of β‐O‐4 ether over the summed up structural units (S+G).

The HSQC spectra of CEL (Figure 4a) showed that the predominate interunit linkages were β‐aryl ether linkage (β‐O‐4, AT), resinol (β‐β, C), and phenylcoumaran (β‐5, B), which were accounting for 64%, 12%, and 3%, respectively. Compared the spectrum of CEL in Figure 4a with lignin extracted using different l‐cysteine additions in Figure 4b–f, the contents of interunit linkages and functional groups changed obviously.

Referring to the previous study, new structures of phenylglycerol structure (Gly) and stilbene structure (SB1, SB5) appeared after native lignin was treated under the certain conditions.^[^ ^40^ ^]^ But the contents were very low based on the resolved structures. In the aliphatic side chain region, the contents of resinols (β‐β) were reduced, while the significant increment of β‐O‐4 including Aox, A, and A_LCL_ could be observed obviously from 2.90% to 25%. Additionally, the contents of condensed S units were about 56% (control group), 36% (CL‐1), 30% (CL‐2), 23% (CL‐3), and 10% (CL‐4), respectively. It suggests that modification of lignin by l‐cysteine, to a certain degree, suppresses the lignin condensation during extraction process.

**Figure 6:**
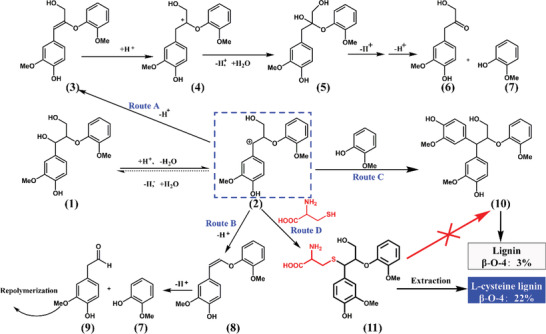
The proposed reaction route of lignin model compound (with β‐O‐4) treated with l‐cysteine.

In order to further explore the structural alternation of lignin during l‐cysteine‐assisted isolation process, a β‐O‐4 dimeric model compound was used to reveal the reaction mechanism (Figure S1, Supporting Information). After reacting with l‐cysteine, the signals of C_β_/H_β_ and C_γ_/H_γ_ in β‐O‐4 did not change significantly, so did the signals of C_1_/H_1_ and C_2_/H_2_ in l‐cysteine. However, a new signal appeared at C_δ_/H_δ_ 52.11/4.2, corresponding to Cα/Hα of l‐cysteine‐substituted β‐O‐4, and the new structure was determined as compound **11** in **Figure** **6**. Furthermore, the structure could also be clearly observed in the spectra of lignin (Figure S2, Supporting Information).

Therefore, combining the mechanism of the thioglycolate method previously reported^[^ ^33^ ^]^ with the structural characterization of lignin in this paper, the possible mechanism of l‐cysteine in lignin separation could be speculated and is shown in Figure 6. Under acid condition, carbocation intermediate (**2**) formed, which may then be dehydrogenated to aryl enol ether structure (**3**) by route A. After that, the double bond of the enol ether structure attacks H^+^ and forms carbocation intermediate (**4**) again. Subsequently, Hibbter's ketones (**6**) and guaiacol (**7**) were formed through continuous dehydrogenation. On the other hand, benzyl carbocation intermediate (**2**) loses a formaldehyde by route B to form enol ether (**8**), followed by further conversion to form arylacetaldehyde structure (**9**) and guaiacol (**7**). Through route C, the benzyl carbocation intermediate (**2**) reacts with nucleophile, such as guaiacol, to form condensed structure (**10**) of lignin. The three pathways above are currently recognized as possible reaction in the depolymerization of lignin catalyzed by acid.^[^ ^16^, ^42^ ^]^ However, in this study, due to the highly nucleophilic HS^−^ in l‐cysteine, when carbocation intermediate was formed, it could be trapped by l‐cysteine quickly (route D) with a new structure formed (**11**). At the same time, the condensation of lignin (**10**) or other repolymerization reduced. Thus, the retention of β‐O‐4 structure in lignin was increased significantly from 3% to 25%.

### 2.7 Hydrogenolysis of Lignin and Lignin Model Compounds

To evaluate the function of l‐cysteine in preserving β‐O‐4 linkage during extraction procedure and the potential of l‐cysteine‐substituted lignin in monophenolics production, we performed hydrogenolysis on the l‐cysteine‐substituted dimeric model compound and extracted lignin samples under different conditions and compared the yield and selectivity of the monomers. The results obtained from the lignin model compounds experiment are shown in **Table** **5**. With l‐cysteine, GG, SS, and GS lignin dimer models could achieve high monomer yields of 26.4%, 28.4%, and 26.6%. While without l‐cysteine, the yields were only 4.3%, 5.7%, and 5.6%, respectively.

**Table 5:** Hydrogenolysis of β‐O‐4 dimeric model compounds

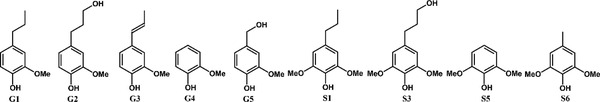										
Dimer	Monomer									Total yields [%]
	G1	G2	G3	G4	G5	S1	S3	S5	S6	
β‐O‐4 (GG)	13.7	6.0	1.6	5.1	–	–	–	–	–	26.4
Control 1	2.0	0.6	–	1.7	–	–	–	–	–	4.3
β‐O‐4 (GS)	–	14.3	–	–	–	–	–	–	14.1	28.4
Control 2	–	–	–	–	5.1	–	–	–	0.6	5.7
β‐O‐4 (SS)	–	–	–	–	–	5.3	3.8	17.5	–	26.6
Control 3	–	–	–	–	–	–	–	5.6	–	5.6

**Figure 7:**
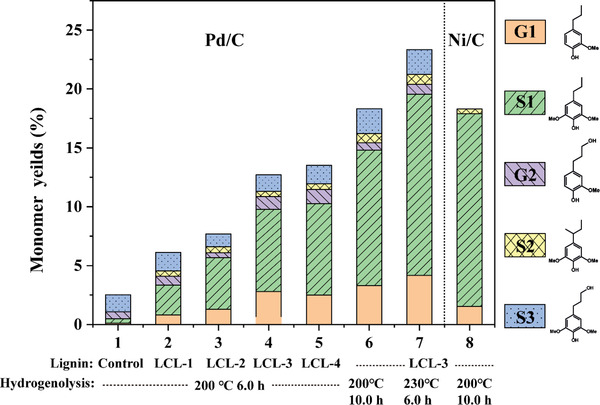
Monomer yields after hydrogenolysis of lignin under different conditions.

As for the lignin, the results are shown in **Figure** **7**, and their selectivity is distributed in Table S1 in the Supporting Information. As a comparison, the reductive cleavage fractionation of *eucalyptus* was performed over Pd/C at 200 °C, and the monomer yield was 43.5 wt% with 92% selectivity to propanolsyringol and propanolguaiacol. The monomer yield of the l‐cysteine‐substituted lignin was 23 wt% over Pd/C at 230 °C for 6.0 h, which was over nine times higher than then controlled experiment, but still lower than the yield from reductive cleavage fractionation (RCF) process. This yield would be even lower when considering the yield of isolated lignin. Nevertheless, this method provides a potential way without using hazard chemicals to fractionate lignin while protecting the aryl ether linkages. Further studies are needed to optimal the process to improve the yield of isolated lignin and the yield of aromatic monomers in the downstream depolymerization process. Hydrogenation of l‐cysteine‐substituted lignin produced only 15% selectivity to propanolphenols over Pd/C, much lower than that of RCF process using the same catalyst.^[^ ^43^ ^]^ This can be explained by a previous study showing that some minor units and carbohydrates effected the product distributions; the isolated lignin without polysaccharide constituents tended to produce more propylphenols and less propanolphenols.^[^ ^44^, ^45^ ^]^

## 3 Conclusions

In this study, an l‐cysteine‐assisted method was developed for lignin separation. The l‐cysteine‐substituted lignin contained β‐O‐4 linkages four times higher than the lignin without l‐cysteine. Model compound study revealed that the l‐cysteine functioned as a stabilizer to react on the Cα in β‐O‐4 linkages. Besides, the S/G ratio of the LCL was also improved. It was also found that the content of side chain hydroxyl group and carboxyl group in LCL was increased by 58.8% and 60.9%, respectively. In addition, the molecular weight of LCL was much lower. The l‐cysteine‐substituted lignin generated 23 wt% of monophenolics by hydrogenolysis, which was also nine times higher than the control sample did. These results suggested that the degradation and condensation of lignin, to some extent, can be inhibited by adding l‐cysteine during the two‐step acid/alkali separation. However, the content of aryl ether linkages was still less than the those in native lignin and further study is required to optimize the extraction procedure and reaction condition to retain more aryl ether linkages. Nevertheless, this study developed a green method for lignin isolation without using harsh condition, toxic chemicals, and organic solvents, and meanwhile being able to retain the aryl ether structures in lignin. This would be interesting and benefiting to the lignin isolation and valorization in future study.

## 4 Experimental Section

### Materials

All the chemicals used in this study were purchased from Macklin Biochemical Co., Ltd. (Shanghai, China). *Eucalyptus* wood chips were provided by Yunnan investment forestry and pulp (Jinggu, Yunnan). The wood chips were cut, grounded, and sieved to collect particles with 40–60 mesh. Then, the grounded wood powder was dewaxed dried in the oven at 60 °C for over 24 h.^[^ ^46^ ^]^ The extractive‐free eucalyptus powder contained 40.1% glucan, 14.1% xylan, 25.1% Kalson lignin, and 1.5% ash, determined by the National Renewable Energy Laboratory (NREL) standard method.^[^ ^47^ ^]^

### Lignin Isolation

Figure S7 in the Supporting Information shows the schematic component separation of lignocellulosic biomass with the assist of l‐cysteine. A dilute acid solution was prepared by adding different volume of HCl solution (8.33, 6.25, 4.17, 2.08, and 0 mL of 37% HCl) and l‐cysteine hydrochloride monohydrate (instead of using pure l‐cysteine) (0, 4.39 g/24.71 mmol, 8.78 g/49.99 mmol, 13.17 g/74.99 mmol, and 17.56 g/100 mmol) into 500 mL of deionized water, and the HCl concentration was 0.2 mol L^−1^. 5 g of oven dried wood powder and 50 mL of the pretreatment solution were added into a 100 mL hydrothermal reactor and sealed. The reactor was kept at 150 °C for 90 min with continuous stirring.

After reaction, the reactor was cooled to room temperature immediately with cooling water, and the reaction mixture was filtered. The solid residue was washed with deionized water until the filtrate was neutral, and then dried at 60 °C in the vacuum oven. The oven dried solid residue was then treated with 0.5 mol L^−1^ NaOH solution at 1:10 solid‐to‐liquid ratio for 120 min at 60 °C and was kept stirring. After treatment, the mixture was cooling down and filtrated. Both the solid residue and filtrate were collected, and the residue was washed with deionized water till neutral and dried at 60 °C in the vacuum oven. The filtrate was acidified by 1 mol L^−1^ HCl solution till the pH ≈ 3, and then was kept in the fridge (4 °C), overnight. The precipitated lignin was collected by centrifugation, and washed by deionized water. Then, the crude lignin was added in 98% dioxane–water solution (1:40, solid‐to‐liquid ratio) at room temperature in the dark and was kept stirring for 8 h. The supernatant was collected by centrifugation and then concentrated by rotary evaporation at 40 °C. The resulting solid was then dissolved in 98% v/v acetone–water solution, and the solid‐to‐liquid ratio was 1:2 to 1:3. Then, the precipitates were formed by slowly adding the solution into 1000 mL of deionized water. Centrifugation and lyophilization were applied to get the purified lignin powder. According to the treatment conditions, the LCL samples were named as LCL‐1 to LCL‐4.

### Synthesis of Lignin Model Compound

Model compounds guaiacyglycerol‐β‐guaiacyl ether (GG, β‐O‐4), syringylglycerol‐β‐syringyl ether (SS, β‐O‐4), and benzylated glycerol‐β‐(4‐methyl syringal) aryl ether (GS, β‐O‐4) were synthesized as previously described.^[^ ^48^ ^]^

### L‐Cysteine‐Assisted Two‐Step Acid/Alkali Treatment of GG, SS, or GS Model Compound

GG (30 mg, 93.75 µmol), SS (30 mg, 78.92 µmol) or GS (30 mg, 66.05 µmol) β‐O‐4 model compound was added into a 25 mL glass vial with 500 µL of 1,4‐dioxane, and then 10 mL dilute acid stock solution (prepared by l‐cysteine hydrochloride monohydrate (13.17 g, 74.99 mmol) and HCl (2.08 mL of 37% HCl) added to 500 mL deionized water) was added to the glass vial. For the control experiment without l‐cysteine, 10 mL of HCl (0.2 mol L^−1^) was added. The reaction vial was sealed and kept at 150 °C for 90 min with continuous stirring. Then, 300 mg of NaOH was added in and reacted for 120 min at 60 °C, kept stirring. After reaction, the resultant mixture was transferred for hydrogenolysis.

In order to further study the role of l‐cysteine in lignin separation, another GG dimer (30 mg, 93.75 µmol) was treated in the same way as above, except the condition of dilute acid pretreatment was kept at 100 °C for 30 min. After reaction, the mixture was transferred into a separating funnel and pH was adjusted to 3 with 1 mol L^−1^ HCl, and then extracted with EtOAc (10 mL × 3 times). The aqueous phase was neutralized by NaOH and collected separately. Then aqueous phase was concentrated at 60 °C under reduced pressure using a rotary evaporator. The solid residue was dissolved by adding acetone, filtered to remove acetone, and concentrated under reduced pressure. In the last, 0.5 mL DMSO‐*d*_6_ was added to dissolve the aim product, and then transferred to an NMR tube.

### Hydrogenolysis of Isolated Lignin and Model Compounds

When isolated lignin was used as the feedstock, 150 mg of l‐cysteine‐substituted lignin (LCL‐1–4) and 150 mg of blank control lignin (Control) was added into a 100 mL high‐pressure Parr reactor (NS200‐P5‐T3‐SS1‐SV), respectively. Then 30 mL ethanol/water solution (80%, V/V) and metal catalyst were also added to the reactor. After stirred and dispersed with ultrasound, the reactor was installed and placed on a heating unit equipped with a magnetic stir bar. To expel the air in the reactor, the reactor was purged with H_2_ (three times) and then pressurized to 40 bar, and then heated up to the desired temperature controlled and kept by the PID temperature controller and stirred with a magnetic stirrer (300 rpm). After the reaction, the mixture was cooled to room temperature and filtered with a G_4_ sand core funnel. Then the aqueous phase was collected for analysis. During the experiment, the amount of catalyst used was 100 mg Pd/C (5% Pd), or 100 mg Ni/C (5% Ni).

When lignin model compounds were used as the feedstock, the resultant mixture was neutralized by 1 mol L^−1^ HCl, dried under reduced pressure using a rotary evaporator. Then it was dissolved in 30 mL ethanol/water solution (80%, V/V), centrifuged at 8000 rpm, and transferred to a 20 mL of supernatant liquor into the reactor with 30 mg Pd/C (5 wt%). The remaining procedure was performed as described above.

As a comparison, lignocellulosic biomass was used as the feedstock. 1 g of biomass and 20 mL of methanol were added to the Parr reactor alone with 200 mg of 5% Ni/C and reacted at 200 °C for 4 h with H_2_ pressurized to 30 bar. The remaining procedure was performed as described above.

### Analytical Methods

The chemical composition of solid residues collected from the two‐step method of eucalyptus was measured according to the method of National Renewable Energy Laboratory (NREL).^[^ ^47^ ^]^

An elemental analyzer (Vario EL cube, Elementar, Germany) was used to analyze the element composition of lignin. Specifically, 2–5 mg of each lignin sample was weighed accurately in silver, wrapped and placed in the tank of elemental analyzer, and fully burned with excess oxygen to determine the content of C, H, N, and S.

GPC (Agilent 1100, USA) was used to analyze the molecular weight of lignin. Specifically, 5 mg of lignin sample was acetylated in 2 mL pyridine‐acetic anhydride (1:1, v/v) in dark for 12 h at room temperature. After finished, the residual pyridine and acetic anhydride were removed thoroughly by evaporation under vacuum. The acetylated lignin was dissolved in 2.5 mL of tetrahydrofuran, and filtrated with 0.22 µm microporous membrane before injected to GPC. Polystyrene samples were used as standard to establish a standard curve for the molecular weight measurement.

A thioacidolysis method was used to measure the monomer yield and the S/G ratio (syringyl/guaiacyl).^[^ ^46^ ^]^ The ^31^P NMR (AVANCE III HD 600, Bruker, Switzerland) was applied to quantify the content of hydroxyl group and carboxyl group of lignin.^[^ ^39^, ^49^ ^]^ The different hydroxyl group contents were calculated by peak integration in Bruker's Topspin 4.1.1

HSQC NMR (AVANCE III HD 600, Bruker, Switzerland) was applied to characterize the interlinkages of lignin. The central solvent peak of DMSO (δ_H_/δ_C_ 2.49/39.5) was used as internal reference. The characteristic signals of various structures were processed by integration in Bruker's TopSpin 4.1.1 software.

Gas chromatography–mass spectrometry (GC‐MS, GCMS‐TQ8040, Shimadzu, Japan) equipped with an SH‐Rxi‐5Sil chromatographic column was used to perform the qualitative analysis of the hydrogenolysis produced monomers. Specifically, 100 µL of an internal standard solution (15 mg 4,4′‐ethylenebisphenol, EBP in 10 mL 1,4‐dioxane) was added to 1 mL of hydrogenated mixture (Section 2.4), followed by rotary evaporation under reduced pressure at 40 °C to remove ethanol/water. Then 3–5 mL of ethyl acetate was added to dissolve and transfer it to a separation funnel. Then 1 m HCl was used to adjust the pH < 3. The aqueous was extracted with ethyl acetate (3 × 2 mL). The organic fractions were combined, washed with saturated ammonium chloride, and dried with anhydrous magnesium sulfate. Then, the organic solvent was filtered and evaporated under reduced pressure at 40 °C. 1 mL of ethyl acetate was added to dissolve again, and then 150 µL of pyridine and 150 µL of silylation reagent (N,O‐bis(trimethylsilyl)trifluoroacetamide, BSTFA) were added. The mixture was reacted in an oven at 50 °C for 40 min. After the reaction, the solution was analyzed with GC‐MS at the following conditions: injection temperature at 250 °C, a split ratio at 20, a column temperature at 50 °C for 1 min; 15 °C min^−1^ to 300 °C, and 300 °C for 7 min. GC‐FID (Nexis GC‐2030, Shimadzu, Japan) equipped with an SH‐Rx1‐1ms chromatographic column was used to perform the quantitative analysis of the monomers. Samples were treated with the same method referred to the GC‐MS analysis. And the monomer yield was calculated based on an internal standard and the effective carbon number (ECN) method.

## Conflict of Interest

The authors declare no conflict of interest.

## Supporting information

Supporting information

## Data Availability

The data that support the findings of this study are available in the supplementary material of this article.

## References

[1] M.‐F. Li , Y.‐M. Fan , F. Xu , R.‐C. Sun , X.‐L. Zhang , Ind. Crops Prod. 2010, 32, 551.

[2] N. Siddique , M. Suzue , M. Kato , K. Hiromori , N. Shibasaki‐Kitakawa , Fuel 2021, 289, 119884.

[3] W. Yang , A. Sen , ChemSusChem 2010, 3, 597. 20437452 10.1002/cssc.200900285

[4] H. Zabed , J. N. Sahu , A. N. Boyce , G. Faruq , Renewable Sustainable Energy Rev. 2016, 66, 751.

[5] K. F. Tzanetis , J. A. Posada , A. Ramirez , Renewable Energy 2017, 113, 1388.

[6] F. Dong , Y. Zhu , G. Ding , J. Cui , X. Li , Y. Li , ChemSusChem 2015, 8, 1534. 25873007 10.1002/cssc.201500178

[7] S. Sun , X. Cao , H. Li , X. Chen , J. Tang , S. Sun , Energy Convers. Manage. 2018, 173, 539.

[8] J. M. Bressanin , B. C. Klein , M. F. Chagas , M. D. B. Watanabe , I. L. de Mesquita Sampaio , A. Bonomi , E. R. de Morais , O. Cavalett , Energies 2020, 13, 4576.

[9] M. V. Galkin , J. S. Samec , ChemSusChem 2016, 9, 1544. 27273230 10.1002/cssc.201600237

[10] S. Paul , A. Dutta , Resour., Conserv. Recycl. 2018, 130, 164.

[11] H. Wang , Y. Pu , A. Ragauskas , B. Yang , Bioresour. Technol. 2019, 271, 449. 30266464 10.1016/j.biortech.2018.09.072

[12] J. S. Kim , Y. Y. Lee , T. H. Kim , Bioresour. Technol. 2016, 199, 42. 26341010 10.1016/j.biortech.2015.08.085

[13] S. X. Li , M. F. Li , J. Bian , S. N. Sun , F. Peng , Z. M. Xue , Bioresour. Technol. 2017, 243, 1105. 28764117 10.1016/j.biortech.2017.07.075

[14] J. Sumphanwanich , N. Leepipatpiboon , T. Srinorakutara , A. Akaracharanya , Ann. Microbiol. 2008, 58, 219.

[15] F. D. C. Siacor , C. F. Y. Lobarbio , E. B. Taboada , Appl. Biochem. Biotechnol. 2021, 193, 1338. 32888162 10.1007/s12010-020-03387-7

[16] M. R. Sturgeon , S. Kim , K. Lawrence , R. S. Paton , S. C. Chmely , M. Nimlos , T. D. Foust , G. T. Beckham , ACS Sustainable Chem. Eng. 2013, 2, 472.

[17] W. Lan , J. S. Luterbacher , Chimia 2019, 73, 591. 31431219 10.2533/chimia.2019.591

[18] X. Zhao , K. Cheng , D. Liu , Appl. Microbiol. Biotechnol. 2009, 82, 815. 19214499 10.1007/s00253-009-1883-1

[19] T. Renders , S. Van den Bosch , S. F. Koelewijn , W. Schutyser , B. F. Sels , Energy Environ. Sci. 2017, 10, 1551.

[20] W. J. Sagues , H. Bao , J. L. Nemenyi , Z. Tong , ACS Sustainable Chem. Eng. 2018, 6, 4958.

[21] L. Shuai , M. T. Amiri , Y. M. Questell‐Santiago , F. Héroguel , Y. Li , H. Kim , R. Meilan , C. Chapple , J. Ralph , J. S. Luterbacher , Science 2016, 354, 329. 27846566 10.1126/science.aaf7810

[22] W. Lan , M. T. Amiri , C. M. Hunston , J. S. Luterbacher , Angew. Chem., Int. Ed. 2018, 57, 1356. 10.1002/anie.201710838 29210487

[23] Z. Chen , W. D. Reznicek , C. Wan , Bioresour. Technol. 2018, 263, 40. 29729540 10.1016/j.biortech.2018.04.058

[24] N. Li , Y. Li , C. G. Yoo , X. Yang , X. Lin , J. Ralph , X. Pan , Green Chem. 2018, 20, 4224.

[25] L. Chen , J. Dou , Q. Ma , N. Li , R. Wu , H. Bian , D. J. Yelle , T. Vuorinen , S. Fu , X. J. Pan , J. J. Y. Jhu , Sci. Adv. 2017, 3, e1701735. 28929139 10.1126/sciadv.1701735 PMC5600535

[26] D. F. Aycock , Org. Process Res. Dev. 2006, 11, 156.

[27] H. Ji , C. Dong , G. Yang , Z. Pang , ACS Sustainable Chem. Eng. 2018, 6, 15306.

[28] D. S. Zijlstra , C. W. Lahive , C. A. Analbers , M. B. Figueirêdo , Z. Wang , C. S. Lancefield , P. J. Deuss , ACS Sustainable Chem. Eng. 2020, 8, 5119.

[29] Z. Zhang , C. W. Lahive , D. S. Zijlstra , Z. Wang , P. J. Deuss , ACS Sustainable Chem. Eng. 2019, 7, 12105.

[30] Z. Sun , B. Fridrich , A. de Santi , S. Elangovan , K. Barta , Chem. Rev. 2018, 118, 614. 29337543 10.1021/acs.chemrev.7b00588 PMC5785760

[31] S. Van den Bosch , S. F. Koelewijn , T. Renders , G. Van den Bossche , T. Vangeel , W. Schutyser , B. F. Sels , Top. Curr. Chem. 2018, 376, 36. 10.1007/s41061-018-0214-3 30151801

[32] B. M. Upton , A. M. Kasko , Chem. Rev. 2016, 116, 2275. 26654678 10.1021/acs.chemrev.5b00345

[33] R. Hatfield , R. S. Fukushima , Crop Sci. 2005, 45, 832.

[34] F. Lu , C. Wang , M. Chen , F. Yue , J. Ralph , Green Chem. 2021, 23, 5106.

[35] J. Bu , Y. T. Wang , M. C. Deng , M. J. Zhu , Bioresour. Technol. 2021, 326, 124751. 33535152 10.1016/j.biortech.2021.124751

[36] C. Mirko , P. Daniela , T. Chiara , G. Giovanni , J. Environ. Manage. 2021, 285, 112098. 33578212 10.1016/j.jenvman.2021.112098

[37] M. Yang , M. Lan , X. Gao , Y. Dou , X. Zhang , Energy Sources, Part A 2019, 43, 1769.

[38] C. Zhao , Z. Hu , L. Shi , C. Wang , F. Yue , S. Li , H. Zhang , F. Lu , Green Chem. 2020, 22, 7366.

[39] Y. Pu , S. Cao , A. J. Ragauskas , Energy Environ. Sci. 2011, 4, 3154.

[40] C. Zhao , S. Li , H. Zhang , F. Yue , F. Lu , Int. J. Biol. Macromol. 2020, 152, 411. 32097737 10.1016/j.ijbiomac.2020.02.241

[41] C. S. Lancefield , O. S. Ojo , F. Tran , N. J. Westwood , Angew. Chem., Int. Ed. Engl. 2015, 54, 258. 25377996 10.1002/anie.201409408

[42] T. Yokoyama , J. Wood Chem. Technol. 2014, 35, 27.

[43] S. Van den Bosch , W. Schutyser , S. F. Koelewijn , T. Renders , C. M. Courtin , B. F. Sels , Chem. Commun. 2015, 51, 13158. 10.1039/c5cc04025f 26086373

[44] Z. Wang , P. J. Deuss , ChemSusChem 2021, 14, 5186. 34398518 10.1002/cssc.202101527 PMC9293178

[45] E. M. Anderson , R. Katahira , M. Reed , M. G. Resch , E. M. Karp , G. T. Beckham , Y. Román‐Leshkov , ACS Sustainable Chem. Eng. 2016, 4, 6940.

[46] F. Yue , F. Lu , R. C. Sun , J. Ralph , J. Agric. Food Chem. 2012, 60, 922. 22191493 10.1021/jf204481x

[47] A. Sluiter , B. Hames , R. Ruiz , C. Scarlata , J. Sluiter , D. Templeton , D. Crocker , Determination of Structural Carbohydrates and Lignin in Biomass: Laboratory Analytical Procedure (LAP), National Renewable Energy Laboratory, Golden, CO 2008.

[48] Y. Liu , G. Lyu , X. Ji , G. Yang , J. Chen , L. A. Lucia , BioResources 2016, 11, 5816.

[49] J.‐L. Wen , S.‐L. Sun , T.‐Q. Yuan , R.‐C. Sun , Green Chem. 2015, 17, 1589.

