# Supplementary material for: Isolation, Characterization, and Depolymerization of l‐Cysteine Substituted Eucalyptus Lignin
Source: Glob Chall. 2022 Mar 3;6(4):2100130. doi: 10.1002/gch2.202100130 (PMC8995711; doi:10.1002/gch2.202100130)
Supplement: Supplementary file 1 — Supporting information [file GCH2-6-2100130-s001.pdf]

Global Challenges
Open Access

Supporting Information

for *Global Challenges,* DOI: 10.1002/gch2.202100130

Isolation, Characterization, and Depolymerization of L-Cysteine Substituted *Eucalyptus* Lignin

Lanlan Shi, Tanhao Zhang, Xin Zhou, Lu Yao, Linjie Yang, Fengxia Yue, Wu Lan,* and Fachuang Lu**

Supporting Information

Isolation, Characterization, and Depolymerization of L-Cysteine Substituted *Eucalyptus* Lignin

Lanlan Shi, Tanhao Zhang, Xin Zhou, Lu Yao, Linjie Yang, Fengxia Yue, Wu Lan*, Fachuang Lu**

L. L. Shi, T. H. Zhang, X. Zhou, L. Yao, L. J. Yang, F. X. Yue, W. Lan, F. C. Lu

State Key Laboratory of Pulp and Paper Engineering, School of Light Industry and Engineering, South China University of Technology, Guangzhou 510640, China

Email: yuefx@scut.edu.cn (F.X. Yue); lanwu@scut.edu.cn (W. Lan); fachuanglu@wisc.edu (F. C. Lu).

F. C. Lu

Department of Biochemistry and Great Lakes Bioenergy Research Center, The Wisconsin Energy Institute, University of Wisconsin, Madison, WI 53726, USA

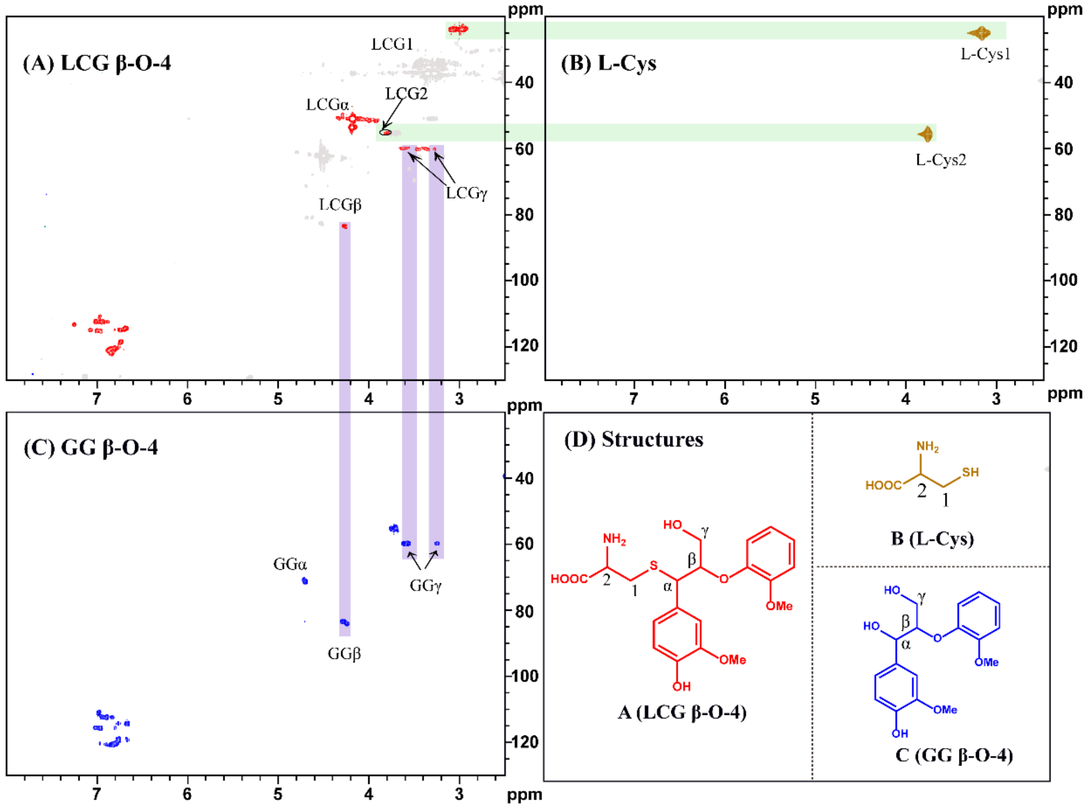

Figure S1. HSQC spectra of (A) the product mixture resulting from the reaction between β-O-4 compounds with L-Cysteine /acid followed by alkali, (B) L-Cysteine, (C) **GG** β-O-4 compounds

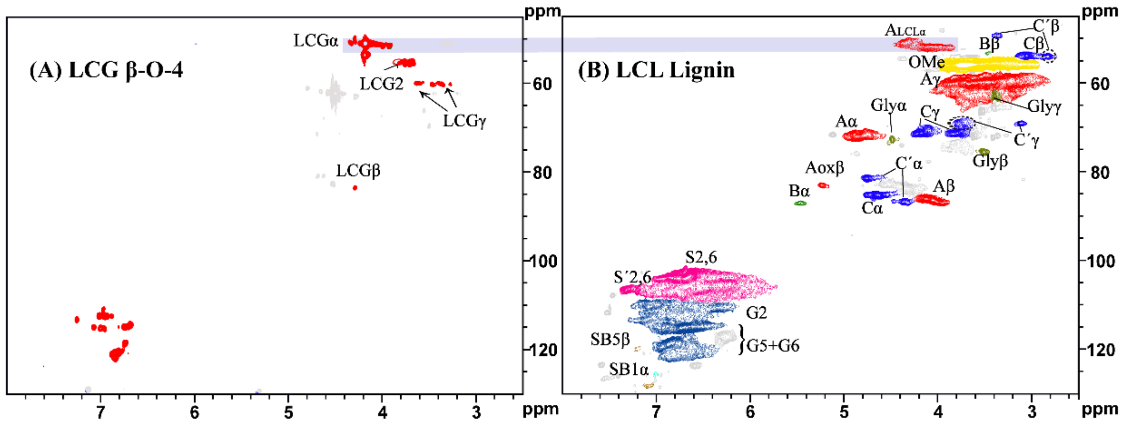

Figure S2. HSQC spectra of the product mixture resulting from the reaction between β-O-4 compounds with L-Cysteine/acid followed by alkali and LCL-3

Table S1. Monomer yields and their selectivity distribution after hydrogenolysis of lignins

Entry	Extraction	Hydrogenolysis conditions	Total Yeilds (wt%)[a]	Selectivity (%)[b]				
				G1	S1	G2	S2	S3
1	Control	200°C, 6h, Pd/C	2.5	4.1	15.0	23.5	-	57.4
2	LCL-1	200°C, 6h, Pd/C	6.1	13.3	41.4	12.1	7.5	25.8
3	LCL-2	200°C, 6h, Pd/C	7.7	16.9	57.0	5.5	6.5	14.0
4	LCL-3	200°C, 6h, Pd/C	12.1	17.7	58.0	9.0	3.8	11.6
5	LCL-4	200°C,6h, Pd/C	13.5	18.5	57.4	8.9	3.7	11.4
7	LCL-3	200°C, 10h, Pd/C	18.3	18.0	62.8	3.4	4.3	11.6
8	LCL-3	230°C, 6h, Pd/C	23.4	17.8	65.8	3.6	3.7	9.0
9	LCL-3	200°C, 10h, Ni/C	18.3	8.3	89.5	-	2.2	-

[a)]The monomer yields were calculated on the basis of Klason lignin. [b)]Selectivity was defined as the yield of the specific monomer over the total monomer yield.

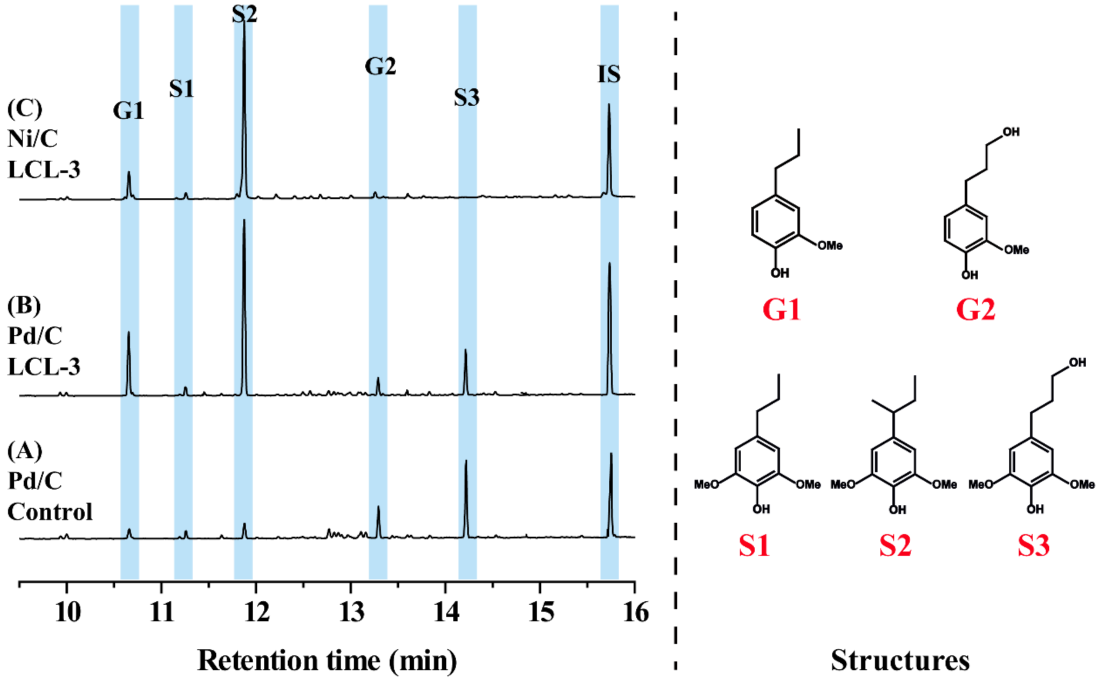

Figure S3. Chromatographic comparison of lignin monomers from hydrogenolysis of lignin in different conditions

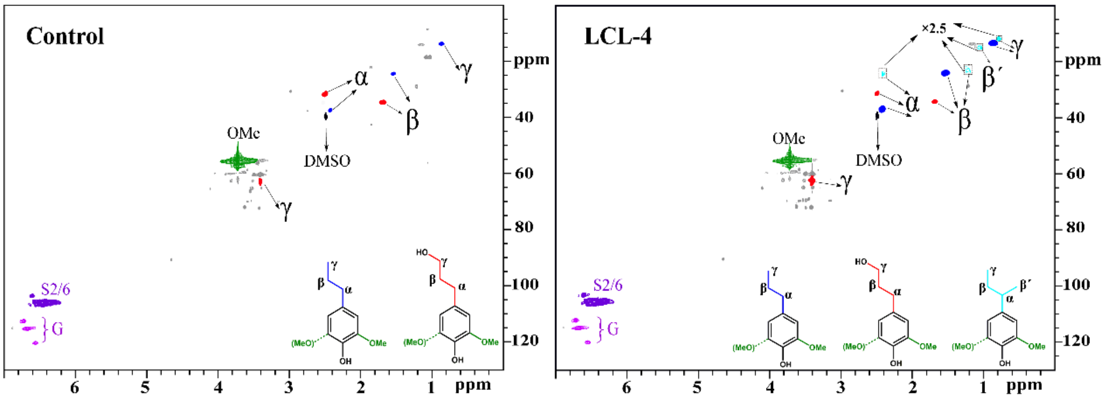

Figure S4. HSQC spectrums of lignin hydrogenolysis products.

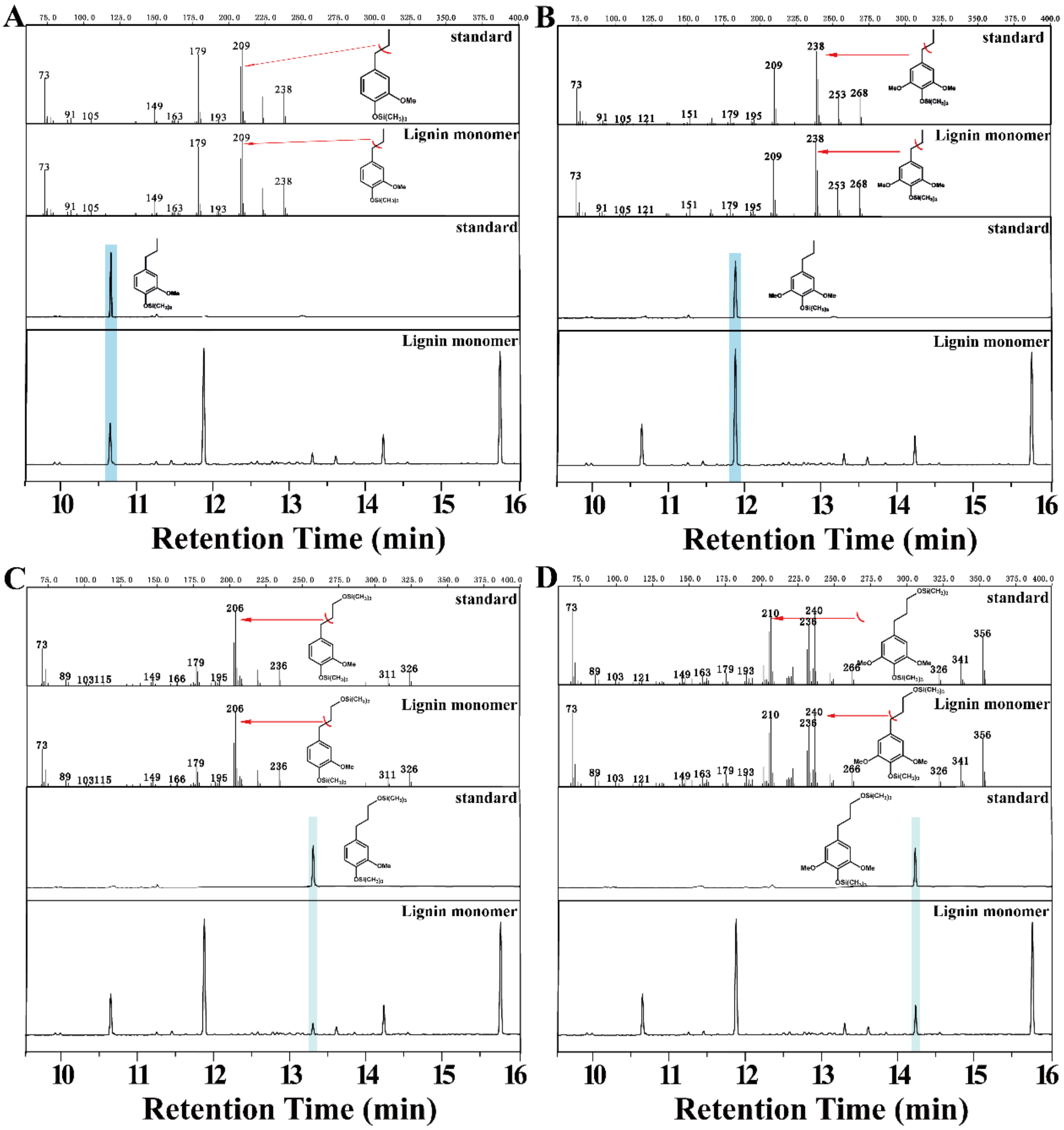

Figure S5. Authentication of monomers produced during hydrogenolysis

Table S2. Increments to calculate the ECN

Atom/group	ECN contribution
Carbon-aliphatic	1
Carbon-aromatic	1
Oxygen-phenol	-1
-C-O-Si(CH_3)$_3$	3.69–3.78

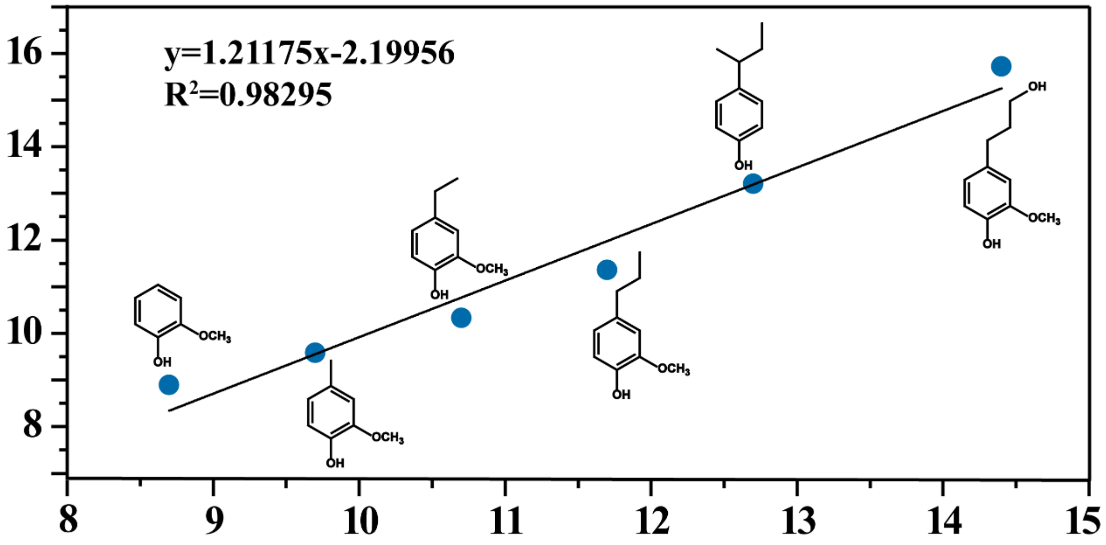

Figure S6. Correction of effective carbon number (ECN) for monomer quantification in EtOAc.

Table S3. Effective carbon number (ECN) calculated based on adjusted ECN rule for lignin monomers

monomer	ECN	monomer	ECN	monomer	ECN
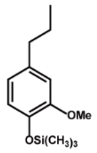	12.0	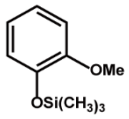	8.3	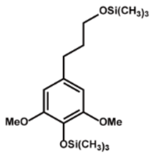	15.2
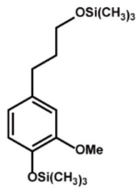	15.2	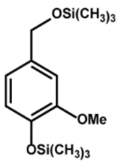	14.0	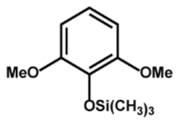	8.3
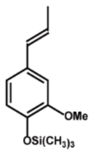	12.0	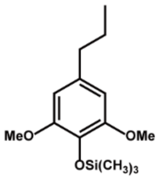	12.0	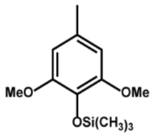	9.6

S2 Chemicals and Materials

All the chemicals used in this study were purchased from Macklin Biochemical Co., Ltd. (Shanghai, China). hydrochloric acid (37%wt), L-Cysteine hydrochloride monohydrate (99%), sodium hydroxide (97%), 1,4-dioxane (99%), sulphuric acid (98% wt), tetrahydrofuran (HPLC), cyclohexanol (>99.0%), chromium acetylacetonate (98%), sodium bicarbonate (99.8%), 2-chloro-4,4,5,5-tetramethyl-1,3,2-dioxaphospholane (TMDP, 98%), ethanethiol (98%), boron trifluoride diethyl etherate (98%), 4,4′-ethylenebisphenol (EBP, >99.9%), ammonium chloride (99.5%), ethanol anhydrous (99.7%), pyridine (99.7%), N,O-Bis(trimethylsilyl)trifluoroacetamide (BSTFA, 98%). dichloromethane (99.7%), nickel (II) nitrate hexahydrate (99.99%), 2-Methoxy-4-propylphenol (98%), guaiacol (>99.0%), hydroconiferyl Alcohol (HPLC), 4-propyl-2,6-dimethoxy (99%), 4-hydroxy-3-methoxyacetophenone (99%), 4-hydroxy-2,6-dimethoxyacetophenone (98%), 2,6-dimethoxyphenol (98%), Anhydrous potassium carbonate (99.7%), N, N-dimethylformamide (DMF 99.5%), benzyl bromide (>98%), pyridinium tribromide (90%), ethyl Acetate (99.7%), hexane (99.7%). Hydrogen (99.999%) was purchased from Guangzhou Guangqi Gas Co., Ltd.

S3 Experimental Analytical methods

S3.1 Schematic component separation of lignocellulosic biomass

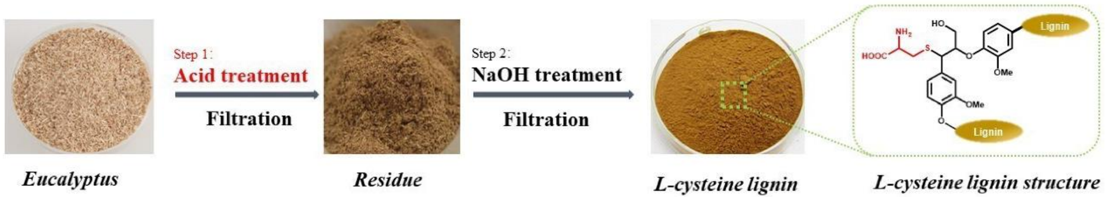

Figure S7. Schematic component separation of lignocellulosic biomass

S3.2 Synthesis of lignin model compounds

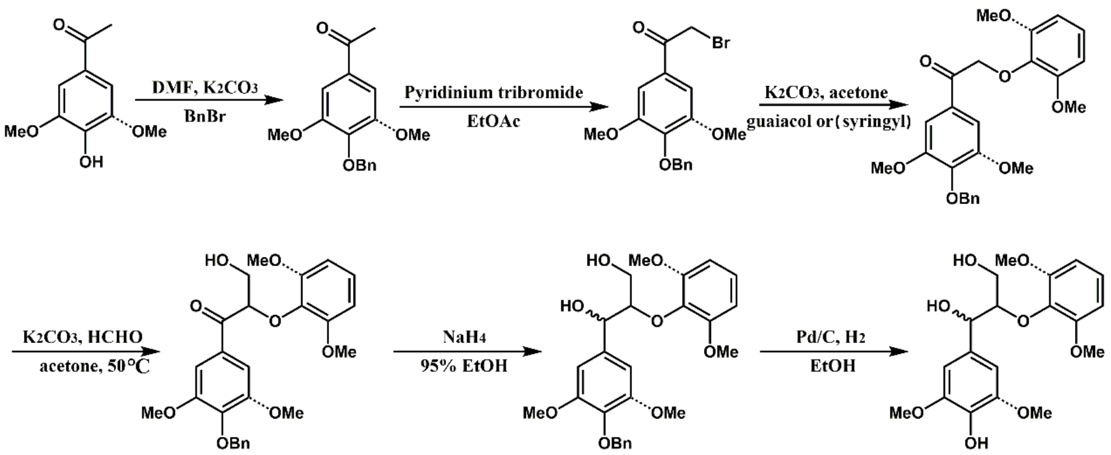

Figure S8. The route of synthesis of lignin model compounds

Model compounds guaiacyglycerol-β-guaiacyl ether (**GG** β-O-4), 4-O-Methyl-syringylglycerol-β-syringyl ether (**SS**) and benzylated glycerol-β-(4-methyl syringal) aryl ether (**GS**) were synthesized as previously described using 4-hydroxy-3-methoxyacetophenone and 4-hydroxy-2,6-dimethoxyacetophenone as a starting material respectively followed the route below. The ^{1}H and ^{13}C NMR spectra of them were shown in **Figure S9**, **Figure S10 and Figure S11.**

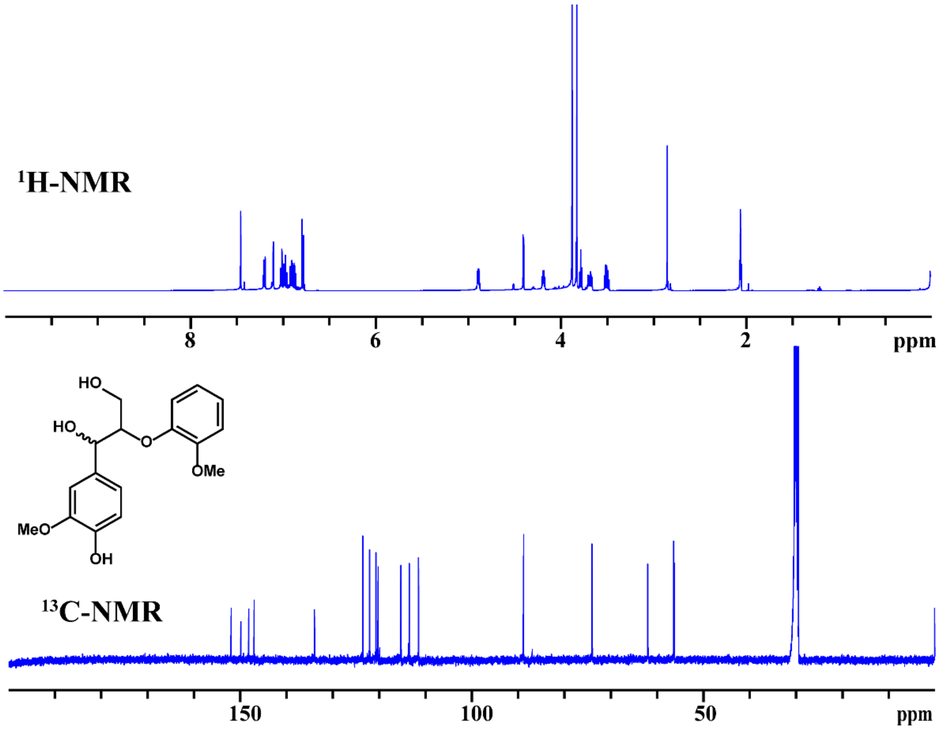

Figure S9. ^{1}H and ^{13}C NMR spectra of GG β-O-4 compound (Acetone-d_6)

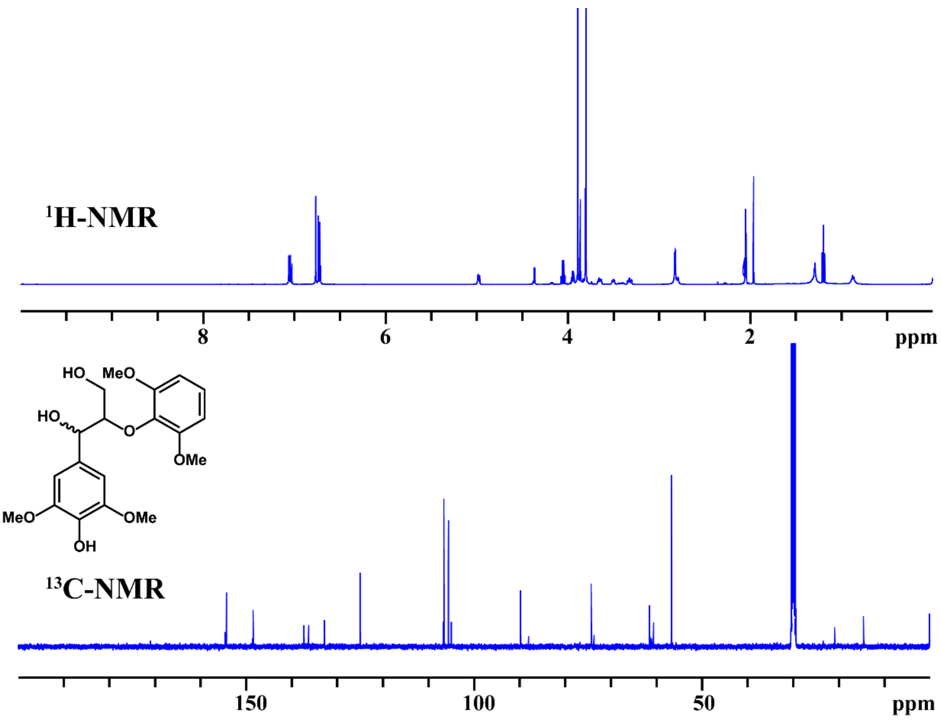

Figure S10. ^{1}H and ^{13}C NMR spectra of SS β-O-4 compound (Acetone-d_6)

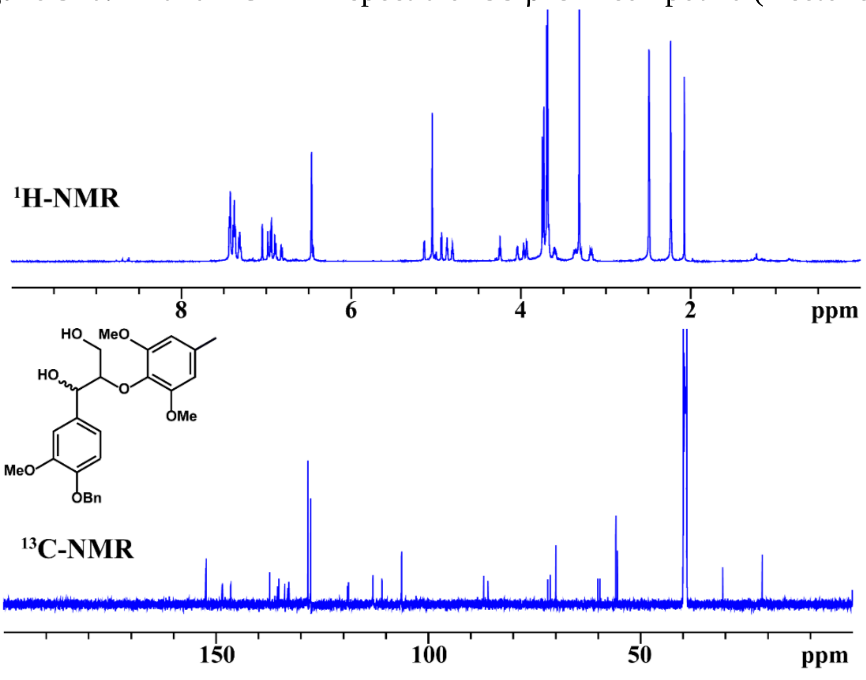

Figure S11. ^{1}H and ^{13}C NMR spectra of GS β-O-4 compound (DMSO-d_6)

S3.3 Analytical Methods

S3.3.1 Analysis of Chemical Composition of Solid Residues

The chemical composition of solid residues collected from the two-step method of eucalyptus was measured according to the method of National Renewable Energy Laboratory (NREL).

Specifically, (0.25–0.50 g) of extracted biomass samples (dried at 105 °C overnight) and added 7.5 mL of 72 wt% H_2SO_4 solution were added to a 20 mL beaker. Then the mixture was left and stirred with a glass rod every 10 min at room temperature for 2 h, followed by transferred it into a round-bottom-flask. Afterward, the glass bottle was filled with 290 mL of water, sealed and heated to 120°C for 1 h in an autoclave. Then, filtered the resultant solution and left the filtrate for sugar analysis by HPLC. For the precipitate, it was washed with water until until the filtrate becomes colorless then dried at 105°C to determine Klason lignin.

HPLC analysis of the sugars was performed with an Dionex ICS-3000 HPLC system equipped with an Agilent 1100 column at 80°C. the mobile phase was water and a flow rate was 0.6 mL/min.

S3.3.2 Thioacidolysis analysis

A thioacidolysis method was used to decomposed and measure the monomer yield and the ratio of syringyl (S) to guaiacyl (G) according to a previous publication. Specifically, 20 mL of distilled dioxane was added to a 25 mL volumetric flask, followed by mixed with 2.5 mL of ethanethiol (EtSH) and 0.7mL of boron trifluoride diethyl etherate (BF_3), and then the thioacidolysis reagent was prepared by diluted the mixture with dioxane to volume. 4.0 mL fresh thioacidolysis reagent and 100 µL of internal standard (13.00 mg/ 60.67 µmol 4,4´-ethylidenebisphenol dissolved in 10 mL dioxane) were added to a 25 ml screw-cap reaction vial, containing 10 mg of lignin. The vial cap sealed, heated to 100 °C and kept for 4 h. After that, the vial was taken out, cooled and transferred into a separatory funnel, washing with CH_2Cl_2 (2 × 2 mL). And then 0.4 M $NaHCO_3$ was used for neutralization, followed by addition of 2 mL 1 M HCl solution to adjust the pH to below 3. Then the mixture was extracted with CH_2Cl_2 (10 mL × 3), and the combined organic phase was washed with saturated NH_4Cl, dried with anhydrous $MgSO_4$, and evaporated under reduced pressure at 40 °C. The residues were silylated in 1 mL CH_2Cl_2, with 100 µL bis(trimethylsilyl)trifluoro-acetamide (BSTFA) and 20 µL pyridine at 50 °C for 40 min, and injected for gas chromatography-mass spectrometry (GC-MS) analysis. The contents of monomer yield and the ratio of syringyl (S) to guaiacyl (G) was calculated as the following calculation (1).

$$RF = (W_S/A_S)/(W_{IS}/A_{IS}) \qquad (1)$$

Where: RF is the response factors of the thioacidolysis monomer standard; W_S is the mass (weight) of the sample, g; W_{IS} is the mass of the internal standard (4,4´-ethylidenebisphenol), g; A_S is the peak area of the sample in the chromatogram; A_{IS} is the peak area of the internal standard. Notice that, RFs of thioacidolysis monomers derived from guaiacyl and syringyl units are depended according to the published report.

S3.3.3 Analysis of ^{31}P NMR

The ^{31}P NMR (AVANCE III HD 600, Bruker, Switzerland) was applied to quantify the content of hydroxyl group (-OH) and carboxyl group (-COOH) of lignin. Specifically, 30 mg lignin (dried in the vacuum drying at 40°C for 24 hours) was weighed and dissolved in 400 μL mixture of deuterated pyridine and deuterated chloroform (1.6:1, v/v) in a 5 mm NMR tube, followed by adding 150 μL mixture of an internal standard substance (4.02 mg/mL cyclohexanol in deuterated pyridine and deuterated chloroform 1.6:1, v/v) and a relaxation regent (3.57 mg/mL chromium(III) acetylacetonate solution in deuterated pyridine and deuterated chloroform 1.6:1, v/v). After that, 100 μL phosphitylating reagent (2-2-chloro-4,4,5,5-tetramethyl-1,3,2-dioxaphospholane, TMDP) was added to the tube. The NMR tube was shaken gently for about 10 min to ensure well mixed and complete reaction. The final mixture was used for ^{31}P NMR analysis.

The different hydroxyl group contents were calculated by peak integration in Bruker's Topspin 4.1.1 as reporteds.

$$C = \frac{(150\times 10-3\rho A_2)/(100.16A_1)}{2M} \qquad (2)$$

Where: C is the content of hydroxyl or carboxyl group, mmol g^{-1}; ρ is the concentration of cyclohexanol, mg mL^{-1}; A_1 is the hydroxyl integral area of cyclohexanol; A_2 is the integral area of hydroxyl or carboxyl groups in lignin structure; M is the sample weight of lignin, g; 150 is the volume of added cyclohexanol, L; 100.16 is the molar weight of cyclohexanol, g mol^{-1}.

S3.3.4 Monomer Identification and Quantification

The monomer identification of hydrogenolysis products was performed by GC-MS and 2D NMR. Quantification analysis was performed by GC-FID and calculated based on the effective carbon number (ECN). The detailed calculation was as follows:

$$n_{EBP} = \frac{W_{EBP\ in\ sample}}{Mw_{EBP}} \qquad (3)$$

$$n_{monomer} = \frac{A_{monomer\ in\ sample}}{A_{EBP\ in\ sample}} \times n_{EBP} \times \frac{ECN_{EBP}}{ECN_{monomer}} \qquad (4)$$

$$W_{monomer} = n_{monomer} \times MW_{monomer} \qquad (5)$$

$$Y_{monomer} = \frac{W_{monomer}\times \mathrm{V}}{W_{Klason\ lignin} or\ W_{extracted\ lignin}} \qquad (6)$$

Where:

n_{EBP} (mmol) is the molar amount of *EBP* in each analyzed sample;

$W_{EBP\ in\ sample}$ (mg) is the weight of *EBP* used as internal standard in each analyzed sample;

Mw_{EBP} (mg mmol^{-1}) is the molecular weight of *EBP*;

$n_{monomer}$ (mmol) is the molar amount of monomer in each analyzed sample;

$A_{monomer\ in\ sample}$ is the peak area of monomer in the GC-FID chromatogram;

$A_{EBP\ in\ sample}$ is the peak area of *EBP* in the GC-FID chromatogram;

ECN_{EBP} is the effective carbon number of *EBP*;

$ECN_{monomer}$ is the effective carbon number of the lignin monomer;

$W_{monomer}$ (mg) is the weight of generated monomer in each analyzed sample;

$MW_{monomer}$ (mg mmol^{-1}) is the molecular weight of monomer;

$Y_{monomer}$ is the yield of monomer based on the weight of Klason lignin;

V (mL) is the total volume of sample, 1 mL of which was used for GC analysis;

$W_{Klason\ lignin} or\ W_{extracted\ lignin}$ (mg) is weight of Klason lignin of the feedstock.
